# Draft Genome Sequence of Hydrocarbon-Degrading *Enterobacter cloacae* Strain S1:CND1, Isolated from Crude Oil-Contaminated Soil from the Noonmati Oil Refinery, Guwahati, Assam, India

**DOI:** 10.1128/genomeA.00367-16

**Published:** 2016-05-12

**Authors:** Arghya Mukherjee, Bobby Chettri, James S. Langpoklakpam, Arvind K. Singh, Dhrubajyoti Chattopadhyay

**Affiliations:** a Department of Biotechnology, University of Calcutta, Kolkata, West Bengal, India; b Department of Biochemistry, North-Eastern Hill University, Shillong, Meghalaya, India

## Abstract

We report here the 4.57-Mb draft genome sequence of hydrocarbon-degrading *Enterobacter cloacae* strain S1:CND1 isolated from oil-contaminated soil in Guwahati, India. S1:CND1 contains 4,205 coding sequences and has a G+C content of 57.45%. This is the first report of the genome sequence of an *E. cloacae* adapted to an oil-contaminated environment.

## GENOME ANNOUNCEMENT

*Enterobacter cloacae* is a Gram-negative, rod-shaped bacterium generally associated with nosocomial infections, and is considered one of the most difficult to treat among the *Enterobacter* sp. (1). Over the years, there have been a few reports of hydrocarbon-degrading *E. cloacae* strains (2), although no genome sequence for any hydrocarbon-degrading *E. cloacae* strain is currently available. *E. cloacae* strain S1:CND1 was isolated from oil-contaminated soil collected from the Noonmati oil refinery in Guwahati, Assam, India. Strain S1:CND1 has been found to degrade alkanes, including n-hexane and n-hexadecane, and polyaromatic hydrocarbon as naphthalene, along with diesel and crude oil. Whole-genome shotgun sequencing was hence carried out to study the genetic constitution and metabolic versatility of this organism. We believe this is the first report of the draft genome sequence of a hydrocarbon-degrading *E. cloacae*.

The genomic DNA for strain S1:CND1 was extracted using an Ultra-Clean Microbial DNA Isolation Kit (MoBio Laboratories, Carlsbad, CA, USA) according to the manufacturer’s protocol. Isolated genomic DNA was then sequenced with an Illumina HiSeq 2500, which generated 4,580,054 paired-end reads. After quality control measures, the reads were assembled using the *de novo* assemblers ABySS v. 3.81 (3), Edena v. 3.130110 (4), MaSuRCA v. 2.2.1 (5), SOAPdenovo2 v2.04 (6), SPAdes v3.1.1 (7), and Velvet v1.2.10 (8). Assembled reads were then integrated using CISA v1.3 (9), which generated 14 contigs with a *N*_50_ length of 445,053 bp and an average length of 326,832.86 bp. The draft genome thus assembled was 4,575,660 bp in length with a G+C content of 57.45% and had 108-fold coverage. Genome annotation for strain S1:CND1 was carried out with the NCBI Prokaryotic Genome Annotation Pipeline, which predicted the presence of 4,205 coding sequences (CDSs), along with 16 rRNAs, 77 tRNAs, 6 noncoding RNAs (ncRNAs), and 50 pseudogenes. Rapid functional annotation for CDSs of strain S1:CND1 was carried out with the RAST annotation server (10), which classified the CDSs into 535 subsystems. Among these, the most abundant subsystems were carbohydrates (*s* = 650 CDSs); amino acids and derivatives (*s* = 467); stress response (*s* = 157); respiration (*s* = 149); fatty acids, lipids, and isoprenoids (*s* = 138); DNA metabolism (*s* = 117); regulation and cell signaling (*s* = 150); protein metabolism (*s* = 168); RNA metabolism (*s* = 150); membrane transport (*s* = 177); virulence, disease, and defense (*s* = 100); cell wall and capsule (*s* = 196); and cofactors, vitamins, prosthetic groups, and pigments (*s* = 253). Genome annotation revealed the presence of hydrocarbon degradation genes as alkane-1-monooxygenase, alkanesufonate monooxygenase, naphthalene 1,2-dioxygenase, and quercetin 2,3-dioxygenase, thus underlining the extensive genetic adaptation of strain S1:CND1 to oil contamination.

A comparison of strain S1:CND1 with genomes in the RAST database identified *Escherichia coli* 88.1467 (score = 501) as its closest neighbor, followed by *E. coli* 88.0221 (score = 490) and *E. coli* 89.0511 (score = 469). *Enterobacter mori* LMG 25706 (score = 344) was identified as the 18th-closest neighbor.

### Nucleotide sequence accession numbers.

This whole-genome shotgun sequencing project for *E. cloacae* strain S1:CND1 has been deposited in DDBJ/EMBL/GenBank under the accession no. LUGN00000000. The version of the whole-genome sequence (WGS) described here is version LUGN01000000.
